# Comprehensive Analysis of Human Epidermal Growth Factor Receptor 2 Through DNA, mRNA, and Protein in Diverse Malignancies

**DOI:** 10.1200/PO.22.00604

**Published:** 2023-07-12

**Authors:** Akram Mesleh Shayeb, Razelle Kurzrock, Jacob J. Adashek, Shumei Kato

**Affiliations:** ^1^ Department of Hematology/Oncology, University of California San Diego, San Diego, CA; ^2^ Medical College of Wisconsin Cancer Center and Genomic Sciences and Precision Medicine Center, Milwaukee, WI; ^3^ Worldwide Innovative Network (WIN) for Personalized Cancer Therapy, Paris, France; ^4^ The Sidney Kimmel Comprehensive Cancer Center, The Johns Hopkins Hospital, Baltimore, MD

## Abstract

**PURPOSE:**

Human epidermal growth factor receptor 2 (HER2) expression (protein immunohistochemistry [IHC] or gene amplification [copy-number variation, CNV]) predicts anti-HER2 therapy responsiveness, although recently it was shown that even low HER2-expressing breast cancers benefit from trastuzumab-deruxtecan. Little is known about HER2 transcriptomic (mRNA) expression, and comparisons between genomic, mRNA, and protein HER2 assays.

**METHODS:**

HER2 status was evaluated using clinical-grade IHC (protein), quantitative reverse transcription polymerase chain reaction (mRNA), and next-generation sequencing (NGS; amplifications).

**RESULTS:**

Multi-institutional HER2 testing was performed on 5,305 diverse cancers including non–small-cell lung (n = 1,175), breast (n = 1,040), and colon cancers (n = 566; N = 3,926 tested for CNV; N = 1,848, mRNA; N = 2,533, IHC). Overall, 4.1% (161/3,926) had NGS *HER2* amplification; 33.3% (615/1,848) had mRNA overexpression; and 9.3% (236/2,533) were IHC-positive. In 723 patients with all three tests (CNV/mRNA/IHC), various amplification/expression patterns occurred: 7.5% (54/723) had all three HER2 tests positive; 62.8% (454/723) had all three tests negative. Discrepant patterns between amplification and overexpression emerged. For instance, 20% (144/723) of patients had mRNA overexpression alone with negative CNV and IHC. A range in values for only mRNA+ cases occurred in different tumor types (eg, 16.9%, breast; 5%, hepatobiliary). There were 53 patients with various tumors from our institution who had all three assays attempted; 22 tested positive for HER2, and seven received anti-HER2 therapy: two patients achieved response: one (esophageal cancer), complete response (≥42 months); one (cholangiocarcinoma), who only had HER2 mRNA positivity (tissue was inadequate for IHC and CNV assessment), partial response (≥24 months) on HER2-based regimens.

**CONCLUSION:**

We demonstrate variability of HER2 (protein and mRNA) expression and amplification using comprehensive assays (CNV, mRNA, and IHC) among diverse cancers. As HER2-targeted therapy indications expand, the relative importance of these modalities merits further evaluation.

## Teaser

Wide variability in HER2 amplification/expression profiles (CNV, mRNA, and IHC) among diverse cohort of malignancies.

## INTRODUCTION

The HER family includes four tyrosine kinase human epidermal growth factor receptors: HER1 (also known as epidermal growth factor receptor [EGFR]), human epidermal growth factor receptor 2 (HER2; ErbB2, CD340, and proto-oncogene Neu), HER3 (ErbB3), and HER4 (ErbB4).^1^ HER2 overexpression results in homodimerization, heterodimerization with other HER family members, autophosphorylation of tyrosine residues within the cytoplasm, and intracellular activation of signaling pathway.^2^ Consequently, HER2 is a proto-oncogene leading to cellular proliferation, tumor invasion, metastasis, and clinically associated with shorter overall survival, higher cancer recurrence, and treatment resistance.^3-6^

**CONTEXT**

**Key Objective**
To determine the correlation between human epidermal growth factor receptor 2 (HER2) gene amplification, mRNA expression, and presence of protein by immunohistochemistry (IHC).
**Knowledge Generated**
The levels of HER2 differ in the same sample between genomic, transcriptomic, and protein levels: 4.1% (161/3,926) of diverse tumors had *HER2* amplification; 33.3% (615/1,848) had mRNA overexpression; and 9.3% (236/2,533) were IHC (protein)-positive. Discrepant patterns between amplification and overexpression emerged; 20% (144/723) of patients had mRNA overexpression alone with negative DNA and protein.
**Relevance**
Seven of 22 patients with clinical correlative data tested positive for HER2; seven patients received HER2-targeted agents, and two responded (esophageal cancer, complete remission ≥42 months; cholangiocarcinoma, partial remission, ≥24 months). The latter patient had only HER2 mRNA positivity (since genomic and IHC assays failed), suggesting that the comparative importance of DNA, RNA, and protein HER2 testing modalities merits further evaluation.

HER2 is overexpressed in 15%-20% of breast and gastric cancers.^7,8^ Targeted therapies against HER2 have shown efficacy against both cancers, with more robust outcomes in breast. For example, trastuzumab, an anti-HER2 monoclonal antibody, improved overall survival in HER2 metastatic^9^ and early breast cancer.^10-12^ Additional anti-HER2 agents were further approved on the basis of outcome improvement, including small molecule inhibitors (eg, neratinib^13^), antibodies (eg, pertuzumab^14,15^), and antibody-drug conjugate (eg, trastuzumab emtansine^16,17^ and trastuzumab deruxtecan^18^).

More recently, studies exploring HER2-targeted therapies have been replicated in other tumors. Indeed, HER2 overexpression is variable by immunohistochemistry (IHC) among multiple cancers, ranging from 2% to more than 50%.^19^ However, a response to anti-HER2 therapies is not uniform^20^ and there are several mechanisms that may explain this disparity. In clinical practice, most widely accepted detection methods for *ERBB2* gene amplification are with fluorescent in situ hybridization (FISH) or with next-generation sequencing (NGS); for HER2 protein expression, IHC is the gold standard. In general, good concordance exists between gene amplification and protein overexpression but this is not always the case.^21,22^ The importance of this disparity is highlighted in patients with discordant gene amplification and protein overexpression; they tend to respond less to HER2-targeted therapies when compared with patients without discordancy.^23^ Second, approximately 5% of breast cancers are designated weak to moderate complete membrane staining observed in >10% of tumor cells under current guidelines from ASCO and the College of American Pathologists:^24,25^ ISH group 2 (HER2/chromosome enumeration probe 17 [CEP17] ratio ≥2.0; average HER2 copy number <4.0 signals per cell), ISH group 3 (HER2/CEP17 ratio <2.0; average HER2 copy number ≥6.0 signals per cell), and ISH group 4 (HER2/CEP17 ratio <2.0; average HER2 copy number ≥4.0 and <6.0 signals per cell).^24,25^ Equivocal cases represent a challenge regarding disease management as an uncertain subgroup with unknown value in predicting benefit from HER2-targeted therapies. However, this group has become more important because of recent results^26^ showing that, in HER2-low (IHC 1+ or 2+ with negative in situ hybridization) metastatic breast cancer, trastuzumab deruxtecan resulted in significantly longer progression-free survival and overall survival than the physician's choice of chemotherapy (perhaps because trastuzumab deruxtecan, in addition to targeting HER2, also carries a payload.) Third, determining HER2 positivity can yield variable outputs between different reference laboratories.^27-29^ Finally, cross-talk between HER2 and other pathways may exist.^30,31^ Consequently, HER2-positive tumors may not have similar responses after anti-HER2 therapies.

Differences in gene expression, protein modification, pathway interactions, and laboratory methodology may be contributing to variable responses from anti-HER2 therapies. Interrogating HER2 at the mRNA regulation may yield additional insights.^32^ One of the advantages of an mRNA assay is that results can be reported in continuous variables instead of categorical scoring variables such as in the case with IHC.^33^ Categorical variables have ceiling effects, have less discrimination between values, and can be subjective depending on the pathologist, particularly in cancers other than breast where standardization is less clear and IHC interpretation is more difficult. Still, little is known about the relationship among HER2 mRNA expression, gene amplification, and protein overexpression in other tumors.

Herein, we report real-world data on the associations between HER2 mRNA, gene amplification, and protein overexpression in a variety of solid tumors.

## METHODS

### Patients

We included all patients with diverse malignancies who were referred to Paradigm Diagnostics, a Clinical Laboratory Improvement Amendments (CLIA) certified, for HER2 amplification/expression status between January 2015 and October 2019. HER2 status was evaluated either by copy-number variation (CNV), mRNA, or IHC testing. A deidentified database with patient cancer diagnosis and testing (CNV, mRNA, and/or IHC) results was available. Treatments and clinical course were evaluated for individuals who were included in the database and were patients at the University of California, San Diego. All investigations followed the guidelines of the Profile-Related Evidence Determining Individualized Cancer Therapy study (UCSD PREDICT study: ClinicalTrials.gov identifier: NCT02478931; IRB-approved) for data collection and any investigational therapies for which the patient gave consent.

### Assays

Formalin-fixed paraffin-embedded specimens were used. The diagnosis of each specimen was confirmed on a freshly cut hematoxylin and eosin–stained slide by a board-certified pathologist. Tissues were micro-/macro-dissected when <20% tumor cells were present (to enrich the sample for tumor). DNA was extracted from all samples, and where feasible, mRNA was also extracted. Complementary DNA was created from mRNA. A proprietary polymerase chain reaction–based method was used to create libraries. All libraries from a given tissue specimen were simultaneously sequenced on an Ion 318 chip on the Ion PGM sequencer (Thermo Fisher Scientific, Waltham, MA). mRNA was analyzed for expression level. CNVs and alterations were reported. A report was generated, reviewed by a board-certified oncologist and pathologist, signed out, and transmitted to the patient's physician. For IHC positivity, the expected stain (HER2/ErbB2 [EP3] Rabbit Monoclonal Antibody) was performed on the membrane (laboratory-developed test [Cell Marque^34^]). IHC cutoff for breast cancer was considered positive if ≥3+ and ≥10% of cells stained. The cutoff for all other cancers was considered positive if ≥2+ with ≥30% of cells stained. For CNV to be considered positive, the measured quantity must have been at least three copies. For mRNA to be considered positive, the measured quantity must be five-fold the expected quantity. All tests were performed in a CLIA-certified laboratory.

### Statistical Analysis

Patient characteristics including systemic therapies were curated retrospectively from review of the charts linking Paradigm pathology reports to University of California San Diego (UCSD) cohort. Patient characteristics, prevalence of HER2 amplification/expression, and associations were summarized by descriptive analysis.

## RESULTS

### Evaluation of HER2 Expression/Amplification Among Diverse Cancers (N = 5,305)

**FIG 1.**
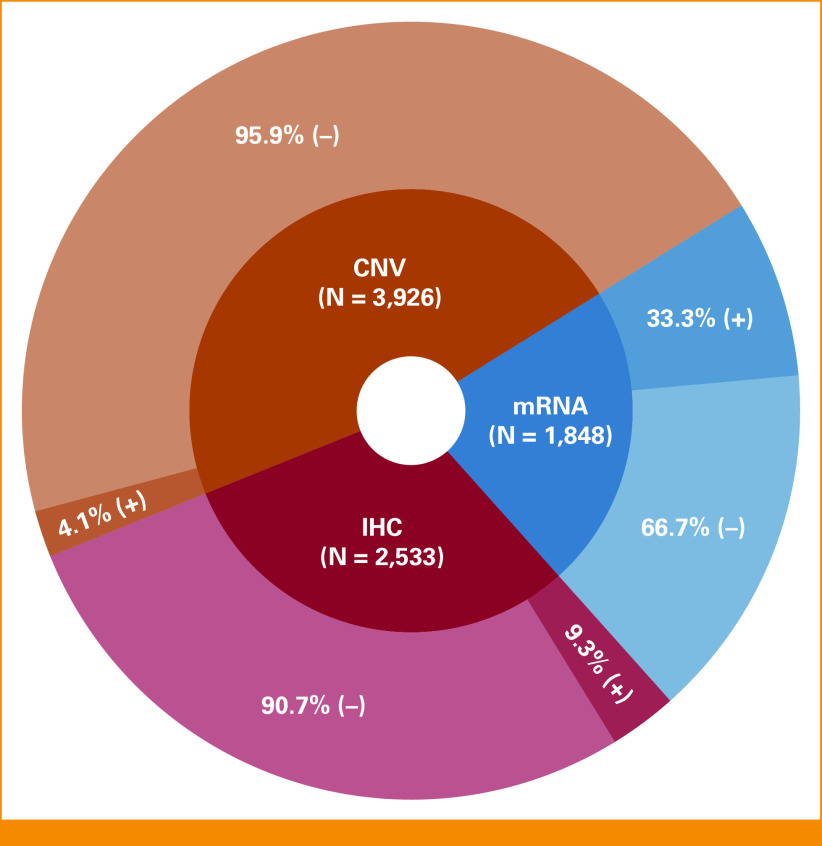
HER2 results per assay (CNV, mRNA, or IHC) among patients with diverse cancers (N = 5,305). Among 3,926 patients who were tested for HER2 CNVs, 4.1% (N = 161) had HER2 amplification. Among 1,848 patients who were tested for HER2 mRNA, 33.3% (N = 615) had high mRNA. Among 2,533 patients who were tested for HER2 IHC, 9.3% (N = 236) were positive. Allowed multiple counts when patients were tested for more than one assay. CNV, copy-number variation; HER2, human epidermal growth factor receptor 2; IHC, immunohistochemistry.

Between 2015 and 2019, HER2 testing was done on 5,305 patients with diverse cancers (N = 3,926 were tested for CNV, N = 1,848 were tested for mRNA, and N = 2,533 were tested by IHC. N = 5,305 had at least one of the HER2 tests. N = 723 had all three testing). HER2 was positive in 4.1% (162/3,926) by CNV, 33.3% (615/1,848) by mRNA, and 9.3% (235/2,533) by IHC (Fig 1). In the 723 patients with all three tests, HER2 was positive in at least one test in 269 patients (37%). The most common diagnosis was non–small-cell lung cancer (n = 1,175), followed by breast (n = 1,040) and colon cancers (n = 566; Data Supplement [Table 1]).

### HER2 Associations in Individuals Who Had All Three Assays (CNV, mRNA, and IHC; N = 723)

**FIG 2.**
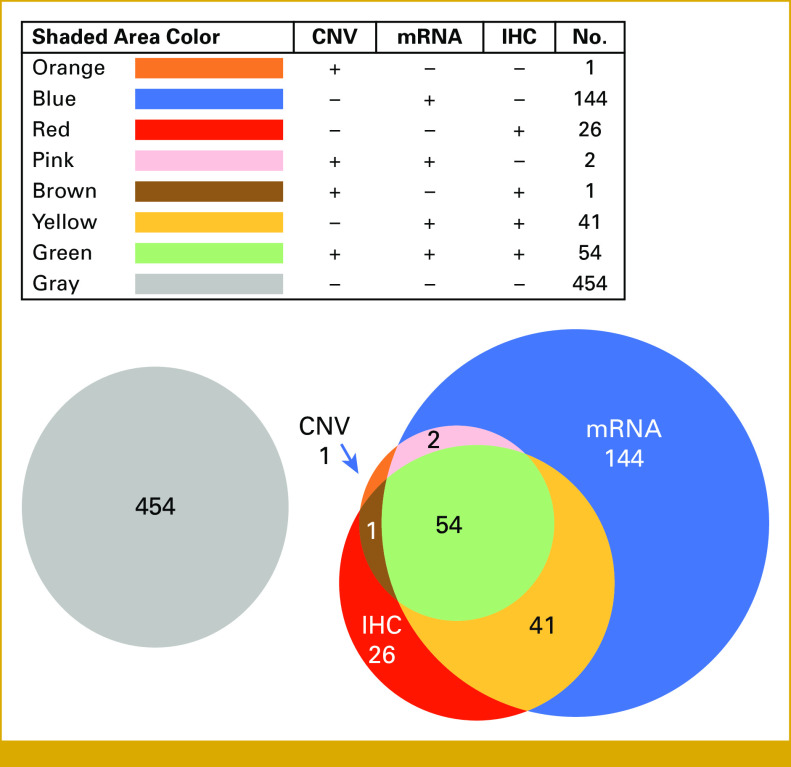
Venn diagram of HER2 positivity for patients who had all three tests performed (N = 723). All patients represented in the Venn diagram had HER2 testing (all three tests including CNV, mRNA, and IHC). The diagram shows the distribution of HER2-positive signaling depending on the assay. The inlet legend describes what each shaded area color represents and the absolute number of patients. For example, the green shaded area includes 54 patients (7.5% of 723 patients) who were positive by CNV, mRNA, and IHC. A total of 454 individuals had negative HER2 testing. CNV, copy-number variation; HER2, human epidermal growth factor receptor 2; IHC, immunohistochemistry.

A variety of amplification/expression patterns were seen (Fig 2). Of 723 patients who had all three tests performed, 7.5% (54/723) of patients had all three HER2 markers positive (CNV [+]/mRNA [+]/IHC [+]). Of 58 patients with positive CNV, 54 had positive IHC and mRNA (93.1%), two cases had positive mRNA but negative IHC, one case had positive IHC but negative mRNA, and one case was negative in both mRNA and IHC. Patients who tested positive for mRNA and/or IHC but negative for CNV were 29.1% (211/723).

We found mRNA overexpression alone in 20% (144/723) of patients with negative CNV and IHC. Of 723 patients who had all three tests done, 33.3% (241/723) were at least mRNA-positive. Only 3.6% (26/723) of patients were IHC-positive when CNV and mRNA were not amplified or overexpressed.

**FIG 3.**
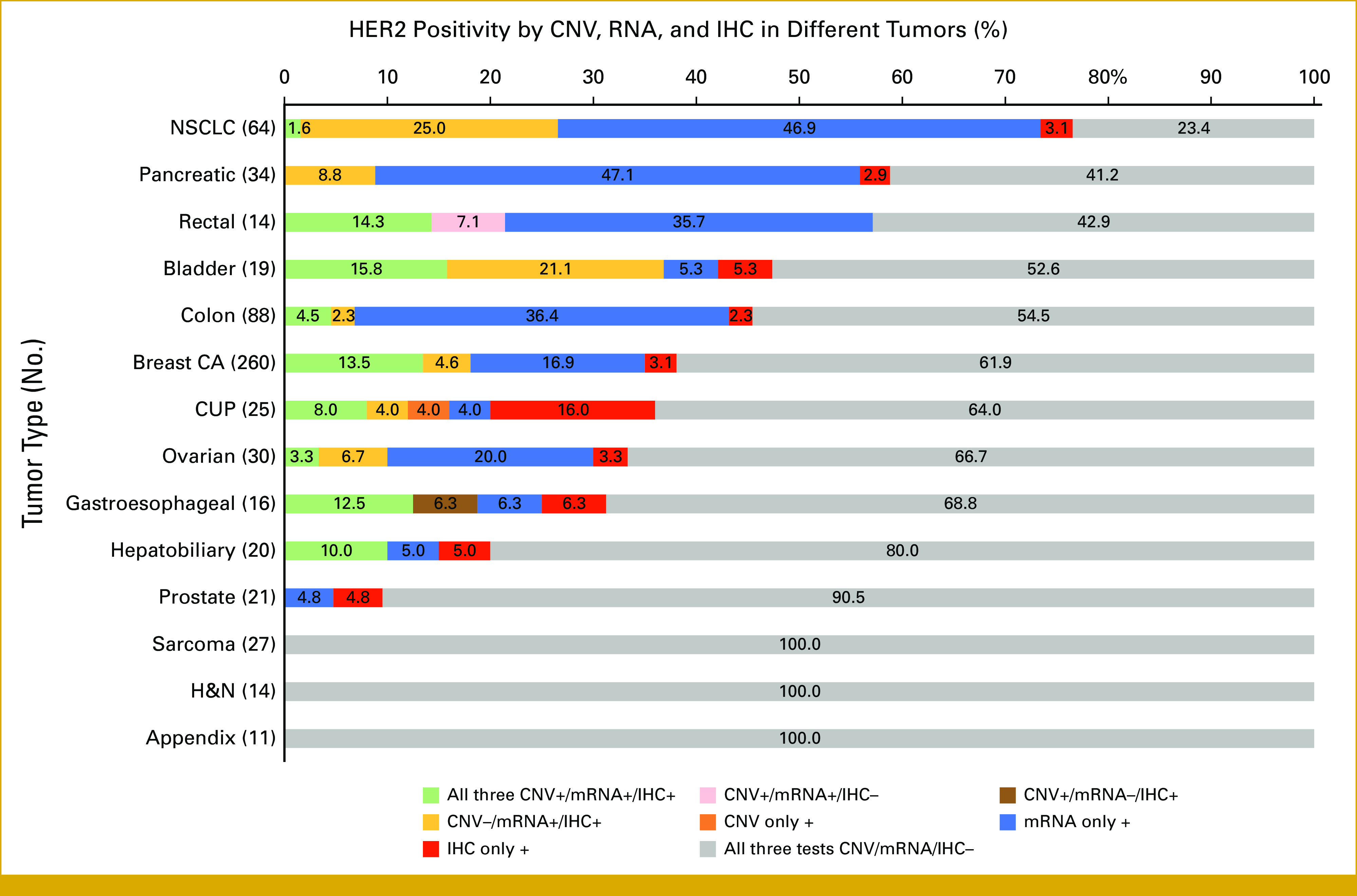
Distribution of HER2 positivity by CNV, mRNA, and IHC per tumor type (N = 723 who had all three assays [CNV, RNA, and IHC] performed; Table 1). Patients who had all three HER2 tests (CNV, mRNA, and IHC) available and tumors with N > 10 are shown in the graph. The bar shows percent of patients depending on the pattern of HER2 positivity. CA, cancer; CNV, copy-number variation; CUP, cancer of unknown primary; H&N, head and neck; HER2, human epidermal growth factor receptor 2; IHC, immunohistochemistry; NSCLC, non–small-cell lung cancer.

Figure 3 shows HER2 expression distribution by cancer type in those patients who had all three assays performed (N = 723). The most common cancer tested for HER2 status was breast (n = 260), followed by colon (n = 88) and non–small-cell lung cancer (NSCLC; n = 64). As mentioned earlier, all three assays were positive in 7.5% of patients (54/723) and at least one of the three assays was positive in 37% of patients (269/723). In breast cancer, HER2 positivity across all three tests was found in 13.5% (35/260), while at least one assay was positive in 38.1% (99/260) of cases. In colon cancer, 4.5% (4/88) showed all three HER2 assays as positive; in NSCLC, 1.6% (1/64); in hepatobiliary, 10% (2/20); in bladder, 21% (4/19); and in rectal, 14.3% (2/14; Table 1).

**TABLE 1.**
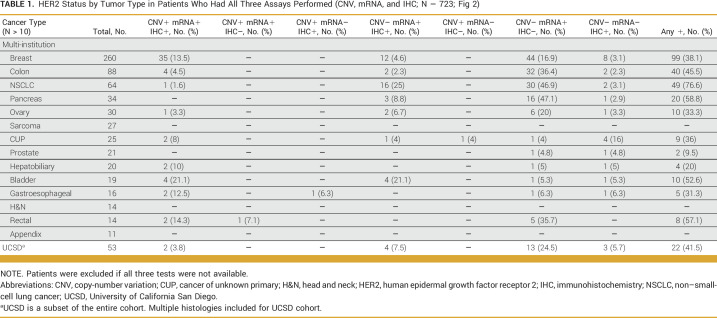
HER2 Status by Tumor Type in Patients Who Had All Three Assays Performed (CNV, mRNA, and IHC; N = 723; Fig 2)

Despite negative CNV and IHC, patients had HER2 overexpression by mRNA in some solid tumors. For example, 47.1% (16/34) of pancreatic cancer, 46.9% (30/64) of NSCLC, and 36.4% (32/88) of colon cancer were HER2 mRNA-positive only. HER2 positivity (by CNV, mRNA, or IHC) was found in 76.6% (49/64) of NSCLC and 58.8% (20/34) of pancreatic cancer. In addition, 36% (9/25) cases with cancer of unknown primary were HER2-positive in at least one test with 88.9% (8/9) cases having CNV and/or IHC positivity (Table 1).

### UCSD Experience With HER2-Positive Tumors (N = 53)

We identified 53 patients with various tumors who had all three assays attempted (CNV, mRNA, and IHC). Two patients had all three assays positive, four patients had CNV–/mRNA+/IHC+, 13 patients had mRNA positive only, three patients had IHC positive only, and 31 patients had all three assays negative (Table 1).

Of 53 patients, 22 tested positive for HER2, and among patients with positive HER2, seven received anti-HER2 therapy. Most patients (5/7) had received at least three previous lines of systemic therapy before initiation of anti-HER2 therapy. Two patients achieved response; one attained complete response and their anti-HER2 treatment is ongoing (Table 2, ID:5, esophageal cancer, positive for HER2 in all three assays [CNV/mRNA/IHC], PFS: ≥42 months); one patient with cholangiocarcinoma was treated with anti-HER2 therapy on the basis of mRNA positivity alone, since CNV and IHC status was unknown because of insufficient tissue quantity; patient achieved partial response with PFS ≥24 months (Table 2; ID:7; Fig 4). An additional patient received anti-HER2 after three lines of therapy, responded in all areas except brain (noting that trastuzumab has poor penetration for blood-brain barrier; Table 2; ID:4, gallbladder cancer, positive for HER2 in all three assays [CNV/mRNA/IHC], PFS: 1.5 months), and passed away.

**TABLE 2.**
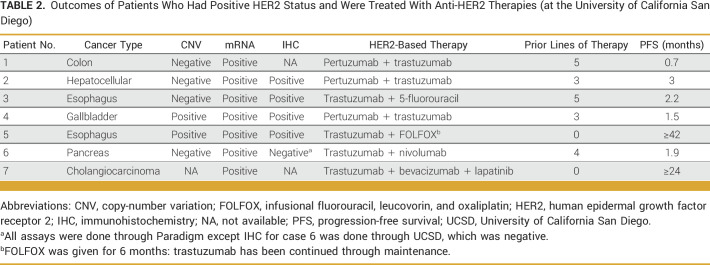
Outcomes of Patients Who Had Positive HER2 Status and Were Treated With Anti-HER2 Therapies (at the University of California San Diego)

**FIG 4.**
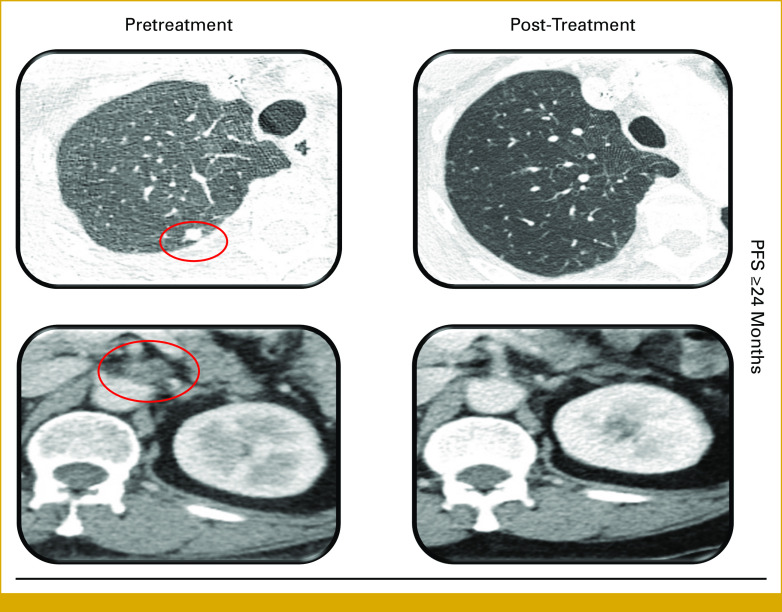
Illustrative case with metastatic cholangiocarcinoma with HER2 overexpression by mRNA assay who achieved durable response with anti-HER2 therapy. A 69-year-old woman with metastatic cholangiocarcinoma who received trastuzumab- and lapatinib-based therapy because of HER2 mRNA positivity and achieved durable partial response (CNV and IHC were inadequate tissue-wise for a read out). The computerized tomographic panels on the left show areas of disease (highlighted by a red circle) before anti-HER2 treatment. The panels on the right show areas of disease improvement 12 months after the patient was treated with anti-HER2 therapy. Specifically, the pulmonary nodule (upper panel) and lymph node (lower panel) have regressed. Thus far, the PFS is ongoing at ≥24 months. CNV, copy-number variation; HER2, human epidermal growth factor receptor 2; IHC, immunohistochemistry; PFS, progression-free survival.

## DISCUSSION

This study highlights HER2 amplification/overexpression patterns, comparing CNV, mRNA, and IHC status across a large cohort of diverse malignancies. Overall, these evaluations establish the wide variability between HER2 assays, from gene amplification, mRNA, to protein level. Importantly, our findings provide novel insight into the frequency of HER2 mRNA expression, which could be targetable with anti-HER2 therapies, as evidenced by our patient, who had advanced cholangiocarcinoma and high mRNA HER2 expression, but insufficient tissue for IHC or copy-number evaluation; this patient has responded to HER2-targeted therapy for ≥24 months.

It is critical to reliably identify molecular alterations that are pharmacologically tractable—HER2 being one of the most important examples. The current standard of care to detect HER2 positivity involve IHC and FISH. However, NGS approaches have been adopted by clinical laboratories because of their short turnaround time and limited biopsy material requirements. Previous studies have shown HER2 CNV assessed by NGS is comparable with FISH and IHC in breast cancer, but concordance is less robust in other malignancies such as stomach cancer.^35^ In our cohort, in breast cancer, HER2 positivity across all three tests (CNV, mRNA, and IHC) was detected in 13.5% of patients (35/260), while at least one assay was positive in 38.1% (99/260) of cases; HER2 IHC was positive (with or without CNV or mRNA positivity) in 21.2% of patients, which is similar to previous reported frequencies.^19,36,37^ Frequencies of HER2 IHC overexpression echoed previous reports for pancreas, stomach, and prostate cancers. Although colorectal cancer and NSCLC had higher IHC overexpression frequencies than previous publications, their NGS CNV amplification was similar. Bladder and CUP had higher CNV amplification and/or IHC overexpression than previous publications.^38^ Discrepancies in expression patterns are likely because of lack of HER2 standardization among non–breast cancer patients, tumor heterogeneity, various HER2 positivity cutoff, and variability between different laboratories.^39^ Furthermore, wide variability of HER2 amplification and overexpression rates is reported in the literature, making comparisons difficult.^19^

In our cohort, HER2 expression by CNV, mRNA, and IHC was negligible in cancers of nonepithelial origin, including bone sarcomas, soft tissue sarcomas, kidney, melanoma, neuroendocrine tumors, and small-cell lung cancers. These findings parallel our previous study where overexpressed HER2 by IHC is primarily found in cancers of epithelial origin.^40^

The molecular information derived by interrogating an individual cancer can help guide treatment. For example, actionable genomic mutations have transformed the management of patients with cancer and improved their outcomes.^41-43^ Unfortunately, not all patients respond, and outcomes for many individuals whose neoplasms harbor actionable genomic targets can still be poor because of resistance mechanisms. Mechanisms for HER2 resistance include obstacles preventing anti-HER2 drugs binding to the receptor; upregulation of HER2 downstream signaling pathways; signaling through alternate pathways; and failure to trigger an immune-mediated mechanism. An alternative explanation to treatment resistance could relate to transcriptomic silencing, including for amplifications, via mechanisms, such as miRNA, alternating RNA splicing, or changes in DNA methylation.^44^ Moreover, in a cohort of 1,879 patients who underwent whole-exome tumor (somatic)/normal (germline) NGS and whole-transcriptome sequencing, 13% of somatic single-nucleotide variants (SNVs) were not expressed as RNA and 23% of patients had at least one or more nonexpressed SNV.^45^ Thus, therapeutic response will likely not occur if the target genomic alteration is not expressed. This phenomenon of transcriptomic silencing highlights the importance of RNA as a complement biomarker tool to NGS. Furthermore, RNA as a potential anti-HER2 biomarker target could provide a useful tool to understand the above resistance mechanisms, improve patient selection that may benefit from anti-HER2 drugs, and predict responses. Biomarker research using RNA has grown substantially in recent years and some studies have shown promise. For example, the WINTHER precision medicine trial demonstrated that transcriptome analysis was feasible and increased the number of patients that could be matched to a targeted therapy compared with genomic analysis alone.^46,47^

Transcriptomics with analysis of mRNA expression may also add value in the field of precision immunotherapy. In an interrogation of 51 markers of the cancer immune cycle, RNA sequencing demonstrated the distinct and complex immune expression patterns of different patients.^48^ Similarly, a simultaneous evaluation of 395 immune-related markers (RNA-seq), 409 exon gene expression, and IHC of program cell death-1 expression in pancreatic adenocarcinoma demonstrated unique immune marker profiles.^49^ These findings suggest the importance of RNA biomarkers to individualize therapy.

Our study has several limitations. First, samples were obtained from different institutions, which could affect discrepancies in expression. Nevertheless, all samples were processed in a single CLIA-certified laboratory, reducing at least the variability of laboratory protocols, antibody used, and interpretation of results. Second, tissue could have been submitted for more advanced heavily pretreated cancers, which could contribute to tumor heterogeneity of expression and selection bias. Third, *ERBB2* (*HER2*) mutations were not reported and could be present in a subset of cancers. *ERBB2* mutations could provide insight into resistance to targeted therapy, and multiple studies are ongoing, evaluating their role for treatment of malignancies. Fourth, there was likely a bias in referrals, with advanced metastatic disease being sent; this is important because findings in metastatic disease may not hold in early-stage disease. Finally, most individuals did not have all three tests (CNV, mRNA, and IHC) performed, restricting association analysis to the most common tumors. Nevertheless, our study included all samples to limit selection bias and reflect real-world experience of the HER2 assay framework as a biomarker in the pan-cancer setting.

In conclusion, our study demonstrates wide variability in real-world HER2 amplification/expression profiles (CNV, mRNA, and IHC) among a diverse cohort of malignancies. Importantly, a patient with advanced cholangiocarcinoma who only had mRNA results (and not genomic or IHC results) and had HER2 transcript expression showed a sustained partial response to anti-HER2 therapy ongoing at 2 years. Discrepancies in HER2 expression between genomics, mRNA, and proteomics require further study to ascertain the comparative predictive value of each assay, and the importance of low expressors *vis a vis* anti-HER2 therapeutics.

## Data Availability

A data sharing statement provided by the authors is available with this article at DOI https://doi.org/10.1200/po.22.00604.
